# Ecological Integrity of Hybrid Ecosystems in the Anthropocene: The Impact of Self‐Organization on Function and Sustainability

**DOI:** 10.1002/ece3.73403

**Published:** 2026-04-08

**Authors:** Elli Groner, Aviva Peeters, Moshe Shachak

**Affiliations:** ^1^ Dead Sea and Arava Science Center Mitzpe Ramon Israel; ^2^ Ben Gurion University Eilat Campus Eilat Israel; ^3^ Negev School of Architecture Shamoon College of Engineering, Architecture Beer Sheva Israel; ^4^ TerraVision Lab, GIS Midreshet Ben‐Gurion Israel; ^5^ Ben‐Gurion University of the Negev, Ecology Midreshet Ben‐Gurion Israel

## Abstract

The Anthropocene is defined by significant human‐driven transformations of Earth's ecosystems, resulting in hybrid systems that merge natural self‐organization with varying degrees of anthropogenic management. These hybrid ecosystems now dominate the terrestrial biosphere, playing a critical role in sustaining biodiversity, ecosystem functions, and human well‐being. In this paper, we argue that self‐organization, the spontaneous emergence of structure and function through local ecological interactions, is a fundamental driver of ecosystem resilience and integrity in hybrid systems. In this manuscript, we synthesize six core self‐organization processes: spatial pattern formation, population self‐regulation, ecological feedbacks, ecosystem engineering, functional food web organization, and functional redundancy, to show their role in underpinning key ecosystem functions across diverse anthrome categories. Using the anthrome framework, we demonstrate how self‐organization operates within croplands, rangelands, village mosaics, forest‐populated mosaics, and seminatural systems, emphasizing the ecological functions these processes support under varying land‐use regimes. Finally, we propose a conceptual framework for assessing ecological integrity in hybrid ecosystems based on the identification of active self‐organizing processes and the mapping of their functional contributions. This process‐based approach offers a practical and scalable tool for monitoring ecosystem health and guiding adaptive management strategies in an era of escalating environmental change.

## Graphical abstract

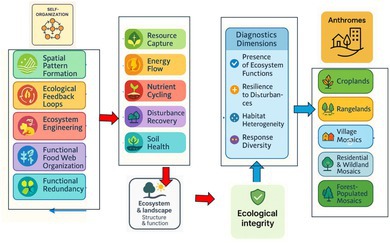

## 1 Introduction

The Anthropocene is an epoch marked by profound and pervasive human impacts on Earth's ecosystems. It features a complex interplay between natural processes and human activities that shape biodiversity and ecosystem functionality (Crutzen 2002; Steffen et al. 2007). This era is characterized by rapid climate change, biodiversity loss, extensive land‐use modifications, and disruptions to global biogeochemical cycles. These transformations significantly influence ecosystem structure and function, and the provision of essential services, including clean air, water, food security, and climate regulation (Steffen et al. 2004).

Currently, most of Earth's terrestrial landscapes can be described as *hybrid ecosystems* shaped by ecological self‐organization and varying degrees of anthropogenic management. These ecosystems exist along a continuum between naturally self‐organized systems, primarily governed by intrinsic ecological processes, and ecosystems predominantly influenced and organized by human intervention (Ellis and Ramankutty 2008).

Globally, hybrid ecosystems now account for more than 75% of the terrestrial biosphere, while minimally affected wildlands account for a mere 22%–25% (Ellis and Ramankutty 2008; Kennedy et al. 2019). The distribution of hybrid ecosystems varies regionally: densely populated cropland‐village mosaics dominate South and Southeast Asia, extensive rangelands and agropastoral systems prevail across Sub‐Saharan Africa, multifunctional agricultural‐residential mosaics are widespread in Europe and North America, and agroforestry landscapes are common in large parts of Latin America and tropical forest frontiers. Indigenous fire and grazing management systems have created resilient hybrid landscapes in Oceania and Australia.

Hybrid ecosystems exemplify the dynamic interaction between self‐organized ecological processes such as feedback loops, succession, and spatial pattern formation, and human‐driven modifications, including land‐use change, resource extraction, and infrastructure development. This complex interplay necessitates a deeper understanding of the role self‐organization plays in the functioning, sustainability, and ecological integrity (EI) of hybrid ecosystems during the Anthropocene.

This paper argues that self‐organization plays a critical role in sustaining ecosystem functions and services in hybrid ecosystems, and that assessing the degree and nature of self‐organization can provide a meaningful indicator of their EI.

An ecosystem that is strongly influenced by human activities while still being governed by natural dynamics should be classified as a hybrid ecosystem. In such systems, the relative influence of bottom‐up (self‐organizing) versus top–down (externally imposed) forces varies. Rather than merely labeling ecosystems as hybrid, it is essential to quantify the relative weight of these forces to better characterize their nature. Some hybrid ecosystems are predominantly self‐organized, with anthropogenic influences largely masked by internal ecological dynamics. Others are primarily shaped by external human forces, while self‐organizing processes are suppressed or obscured. Still, some systems represent a more balanced interaction between the two. This perspective suggests a continuum—ranging from ecosystems driven largely by self‐organization to those dominated by anthropogenic processes.

To systematically evaluate the role of self‐organization in the EI of hybrid ecosystems, we adopted the anthrome framework developed by Ellis and Ramankutty (2008). This framework classifies human‐influenced ecosystems based on population density, land use, and ecological structure. Each anthrome represents a unique configuration of human‐nature interactions, combining human land use with varying levels of natural ecosystem structure and self‐regulation.

These ecosystems are “hybrids” not only in terms of land use but also in their internal ecological dynamics. Even under intensive human management, self‐organizing processes, such as spatial pattern formation, species self‐regulation, feedback loops, and ecosystem engineering, continue to operate and affect ecosystem resilience and functionality (Rietkerk et al. 2004; Murdoch 1994; Jones et al. 1994).

Understanding the role of self‐organization is essential for assessing the sustainability and adaptive capacity of hybrid ecosystems. The following sections explore key aspects of hybrid ecosystem structure and function, first by investigating the role of self‐organization in hybrid ecosystem processes, and then by examining EI, ecosystem services, and specific self‐organizing processes within the anthromes. We conclude by proposing a conceptual framework for navigating self‐organization and EI in the Anthropocene. This framework provides both theoretical and practical foundations for evaluating ecosystem health, guiding sustainable management strategies, and balancing EI with human well‐being amidst rapid environmental change.

## 2 The Role of Self‐Organization in Hybrid Ecosystem Processes

Self‐organization and imposed organization are two distinct but interrelated processes that shape the structure, function, and integrity of hybrid ecosystems. Understanding the specific role of self‐organization in ecosystems influenced by external interventions is crucial for assessing the effects of both ecological and anthropogenic factors on life‐supporting ecosystems.

*Self‐organization* refers to a spontaneous, bottom‐up process in which system components interact independently, creating patterns and structures without external control. This phenomenon is observable in natural ecosystems, such as organization within ant colonies (Gordon 1999) or neural activity (Kelso 1995). Self‐organization exemplifies the inherent adaptability and resilience of ecosystems as they evolve in response to both internal and external changes (Levin 1998).

In contrast, *imposed organization* is a top–down process, in which an external authority designs and controls a system to achieve specific objectives. This approach is typical in human systems, such as agricultural fields, urban ecosystems, planted woodlands, or managed nature reserves, where deliberate interventions are applied to achieve desired outcomes (Cumming 2011).

In the Anthropocene, most ecosystems are hybrids of these two organizational modes. Each ecosystem contains elements that are self‐organized and others that are shaped by anthropogenic forces. In healthy, natural systems, self‐organized dynamics dominate, while in artificial, anthropogenic systems, imposed organization prevails. Each system can be characterized based on the proportion of self‐organized and imposed elements.

A planned orchard, planted in neatly aligned rows, is an example of an imposed organization, while the spontaneous spacing of shrubs in a natural landscape, driven by competition for water and nutrients, illustrates self‐organization. Interestingly, both processes can generate similar spatial patterns, yet they arise from fundamentally different mechanisms (Rietkerk et al. 2004).

In both natural and hybrid ecosystems, self‐organization manifests through six key ecological processes: (1) Pattern formation, which enhances spatial resource capture and resilience; (2) Population self‐regulation, which stabilizes species dynamics; (3) Ecological feedback loops, which reinforce stability or drive adaptation; (4) Ecosystem engineering, which creates critical microhabitats and resource hotspots; (5) Functional food web organization, which maintains the flow of energy and nutrients; and (6) Functional redundancy and response diversity, which buffer ecosystem functions under disturbances. Together, these processes underpin the functioning and resilience of hybrid ecosystems, integrating self‐organized ecological processes with human management. This integration strengthens EI and ensures sustained delivery of ecosystem services in the Anthropocene.

Spatial pattern formation is one of the most observable manifestations of self‐organization in ecological systems. Recurrent spatial arrangements such as shrub patches, vegetation bands, or “fairy circles” commonly emerge in resource‐limited landscapes as a response to feedback between vegetation growth, water redistribution, and soil characteristics. These patterns, which optimize resource capture and reduce ecological stress, are particularly prominent in arid and semi‐arid environments (Rietkerk et al. 2004).

The second fundamental self‐organization process is population self‐regulation, whereby species regulate their own abundance through feedback mechanisms such as resource limitation, predation, and disease dynamics. These processes prevent uncontrolled population booms or crashes, helping stabilize community composition and its ecosystem functions (Murdoch 1994). The concept of carrying capacity (or self‐thinning) represents a self‐organizing mechanism that stabilizes biomass or population levels.

Ecological feedback loops, both positive and negative, further regulate system behavior. Positive feedback reinforces desirable processes, such as vegetation‐driven increase in soil moisture, while negative feedbacks, such as predator control over herbivore populations, counteract excesses and promote system stability. This self‐organized feedback is essential for understanding ecological thresholds, phase transitions, and regime shifts (Walker et al. 2004).

Ecosystem engineering is another key component of self‐organization. It involves organisms that modify the physical environment in ways that affect not only their own survival but also the survival and function of the broader ecosystem. Examples include beavers constructing dams, corals building reef structures, and cyanobacteria forming biological soil crusts that regulate water distribution and soil erosion in drylands (Jones et al. 1994).

Emerging self‐organizing functions of food webs also play a critical role, as the structure and strength of trophic interactions affect energy flow, nutrient cycling, and system stability. Well‐structured food webs exhibit resilience through redundancy and buffering among trophic levels, preventing the collapse of key ecosystem processes, even under perturbation (Thebault and Fontaine 2010).

Lastly, functional redundancy and response diversity describe how multiple species fulfilling similar roles but differing in their sensitivity to environmental change are fundamental to self‐organized resilience. Trait diversity ensures that essential ecosystem functions, such as pollination, decomposition, and primary productivity, are maintained even as species decline (Elmqvist et al. 2003; Mori et al. 2013).

Response diversity plays a central role in maintaining functional stability, despite natural stressors and anthropogenic impacts. In systems such as agroecosystems, managed rangelands, and seminatural forests, species that share similar ecological functions but vary in their tolerance to disturbance help sustain key services during times of stress (Elmqvist et al. 2003; Oliver et al. 2015).

Response diversity enables ecosystems to bridge natural functioning with human needs, ensuring the sustained delivery of services such as pest control, water regulation, and soil stabilization even when certain components of the system are lost. Moreover, response diversity enhances the system's capacity for self‐repair. After disturbances such as drought, fire, or overgrazing, species with differing responses can reestablish functional structures and processes through self‐organization, without extensive human intervention (Mori et al. 2013).

Natural processes within ecosystems reduce the dependence on external input and promote more sustainable forms of land use. In this context, self‐organization acts not only as an ecological principle but also as a practical mechanism for adaptive management, buffering hybrid ecosystems against instability and fostering long‐term sustainability.

Ultimately, self‐organization, reinforced by response diversity, serves as a foundational mechanism through which hybrid ecosystems retain integrity and functionality in the Anthropocene. Acknowledging and enhancing these self‐organizing processes in ecological management can inform more resilient and adaptive strategies for sustaining ecosystem services in an era of unprecedented environmental change.

## 3 Self‐Organizing Processes, Ecological Integrity, and Ecosystem Services of Specific Hybrid Ecosystems: The Anthromes

The Anthropocene has brought about the global dominance of hybrid ecosystems that combine natural self‐organization with varying degrees of human management. The anthrome framework, developed by Ellis and Ramankutty (2008), classifies these human‐influenced systems based on population density, land use, and ecological structure. Building on this framework, it is crucial to understand how self‐organization sustains EI and ecosystem services across different anthrome categories, each representing a continuum between natural processes and human intervention.

Although human activities dominate croplands, self‐organizing processes remain vital. Spatial pattern formation is evident in diversified cropping systems, such as intercropping and agroforestry, which optimize resource use and stabilize yields (Kremen and Miles 2012). Population self‐regulation occurs through predator–prey dynamics between natural enemies and crop pests (Letourneau et al. 2009). Feedback loops, such as soil health improvement through organic matter recycling, enhance fertility and resilience (Gliessman 2015). Plant root systems that enhance soil structure and microbial communities act as ecosystem engineers (Lavelle et al. 1997). Food web organization, by pollinators and decomposers among others, ensures sustained production (Tscharntke et al. 2005). Functional redundancy and response diversity, maintained by cultivating multiple crop varieties with varying tolerances to drought or pests, buffer agricultural systems against climatic variability (Altieri 1999). Agricultural practices become sustainable when self‐organized processes occur, and farmer intervention is minimized. For example, nitrogen supply from a self‐organized food web is considered healthier than a continuous supply of artificial nitrogen. The balance between bacteria and fungi, and the control of harmful pests by self‐organized food webs, reduces the need for pesticides and antibiotics. Rather than categorizing agriculture as either intensive or organic, agricultural practices can be viewed along a gradient of self‐organization levels. Thus, self‐organization can be used as a measure of system health, and the level of EI of the agricultural system depends on the extent of self‐organization present.

In rangelands, self‐organizing vegetation patterns emerge from grazing‐mediated feedback and differential resource availability (Rietkerk et al. 2004; Briske et al. 2023). Population self‐regulation is observed through herbivore‐vegetation interactions that prevent overgrazing under moderate stocking rates (Fuhlendorf et al. 2012). Feedback loops, such as positive feedback between plant cover and soil moisture retention, sustain grassland productivity (Bestelmeyer et al. 2006). Ecosystem engineering by species like prairie dogs and termites alters soil structure, influencing hydrology and plant diversity (Davidson, de Araújo, and Artaxo 2012). Functional food webs involving grazers, predators, and scavengers maintain ecosystem energy flow (Polis et al. 1996). Functional redundancy among different grass and shrub species enhances rangeland resilience to drought and grazing pressure (Sasaki et al. 2009). In village landscapes and rural mosaics, self‐organization is evident in patchy mosaics of gardens, fallow fields, and forests that emerge from decentralized land‐use decisions (Altieri and Toledo 2011). Population self‐regulation is maintained through local seed networks that promote diverse crop varieties adapted to different microclimates (Zimmerer 1996). Nutrient cycling supports feedback loops through composting and integration of livestock into farming systems (Tittonell 2014). Ecosystem engineering is evident in traditional water harvesting infrastructures, which create microhabitats and sustain soil moisture (Reij et al. 1996). Functional food webs, involving native pollinators and pest‐control organisms, emerge naturally in diversified fields (Perfecto and Vandermeer 2010). Functional redundancy is preserved through polycultures and traditional crop rotations, enhancing system adaptability (Altieri 1999).

In residential and wildland mosaics, self‐organization is evident in spontaneous succession processes in vacant lots, parks, and urban peripheries (Aronson et al. 2017; Kowarik 2011). Population self‐regulation operates through urban wildlife dynamics, such as predator–prey interactions among small mammals, birds, and their predators (Shochat et al. 2006). Feedback loops regulate green space resilience; for example, vegetation improves soil permeability and mitigates urban runoff (Bolund and Hunhammar 1999). Tree roots act as ecosystem engineers by stabilizing the soil and providing shade, thereby influencing microclimates (McKinney 2006). Functional food webs develop in urban settings; insects, birds, and mammals facilitate seed dispersal and pest control (Alberti 2005). Functional redundancy in urban flora, with diverse plant species adapted to different disturbance regimes, ensures continued green infrastructure services (Kattwinkel et al. 2011).

In forest‐populated mosaics, spatial patterns form through natural gap dynamics and regeneration after selective logging or small‐scale agricultural endeavors (Chazdon 2003; Chazdon and Guariguata 2016). Population self‐regulation is mediated by density‐dependent seedling survival and herbivory (Connell 1978). Feedback loops involving forest canopy closure and microclimate stabilization help maintain successional trajectories (Pickett and White 1985). Ecosystem engineering by large trees and animals (e.g., seed dispersal by primates or birds) shapes forest structure and biodiversity (Asner et al. 2010). Functional food webs involving multi‐trophic interactions between plants, herbivores, predators, and decomposers maintain nutrient cycling and productivity (Terborgh et al. 2001). Functional redundancy is preserved through species‐rich assemblages that ensure functional continuity despite localized disturbances (Mori et al. 2013).

Seminatural systems, such as rewilded pastures and abandoned fields undergoing secondary succession, are characterized by natural spatial pattern formation, often influenced by disturbance regimes like fire and grazing (Benayas et al. 2009). Population self‐regulation occurs through herbivore browsing pressures that modulate vegetation dynamics (Bond 2019). Feedback loops include vegetation‐fire interactions that stabilize or shift community composition (Pausas and Keeley 2009). Large herbivores, such as bison or horses, are an example of ecosystem engineers that modify grassland structure (Sandom et al. 2014). Functional food webs develop through recolonization by trophic guilds, from primary consumers to apex predators (Svenning et al. 2016). Functional redundancy and response diversity are essential in these systems, increasing resilience to climatic variability (Perring et al. 2015).

Within all anthrome categories, these six self‐organizing processes form the ecological foundation that underpins resilience, EI, and the provision of ecosystem services. Recognizing, protecting, and enhancing these self‐organizing dynamics, particularly through the promotion of functional redundancy and response diversity, offers a pathway toward adaptive and sustainable management of hybrid landscapes in an era of rapid global change.

These examples can be categorized based on ecosystem history, management, self‐organization, and development.

## 4 Ecosystem Types Along a Self‐Organization Gradient

Hybrid ecosystems span a range of configurations defined by varying degrees of autonomous ecological self‐organization and external human influence. These configurations are best understood in terms of feedback dominance, that is, whether ecosystem structure and resilience arise primarily from endogenous ecological regulation or from sustained external inputs. Importantly, however, the ecosystem types identified along this gradient should not be interpreted as simple linear mixtures. Rather, they represent qualitatively distinct regimes that emerge once ecological thresholds are crossed, often accompanied by changes in feedback architecture, reversibility, and adaptive capacity.

Within this framework, we identify four primary ecosystem types: wild systems, novel ecosystems, dialectical ecosystems, and coerced regimes (Table 1). Each type reflects a distinct balance between ecological autonomy and managerial control, with direct implications for sustainability, vulnerability, and EI.

**TABLE 1:** Summary of ecosystem types along the self‐organization gradient.

Ecosystem type	Core properties	Representative examples	References
Wild systems	Dominated by intrinsic ecological feedback; resilience arises from biodiversity, trophic complexity, and endogenous regulation; autonomous self‐organization	Tropical rainforests; Coral reefs; Beaver‐engineered wetlands	Davidson, de Araújo, and Artaxo (2012), Davidson, Detling, and Brown (2012), Hughes et al. (2010), Hood and Larson (2015)
Novel ecosystems	Self‐organizing systems shaped by historical anthropogenic forcing; reorganized around altered baselines with modified feedback; constrained by legacy effects; partial reversibility or ecological lock‐in possible	Postindustrial forests; Abandoned agricultural lands; Urban wildlands	Hobbs et al. (2009), Hallett et al. (2013)
Dialectical ecosystems	Emergent regimes formed through dynamic tensions between ecological self‐organization and human organizational forces; coupled social–ecological feedbacks co‐determine attractor configurations and adaptive trajectories	Adaptive comanaged landscapes; Urban green infrastructures	Angeler and Maybee (2025)
Coerced regimes	Maintained by sustained external inputs; endogenous ecological feedback suppressed or overridden; externally enforced stability; management‐dependent and brittle	Industrial agriculture; Chemically treated eutrophic lakes; Fire‐suppressed forests	Angeler et al. (2020), Sundstorm et al. (2023), Shimoda and Arhonditsis (2016), Kreider et al. (2024)

## 5 Wild Systems

At the high end of the self‐organization spectrum are primarily wild ecosystems, in which internal ecological interactions and feedback loops dominate system dynamics. These systems are typically only lightly managed, and their resilience and stability emerge from biodiversity, trophic complexity, organismal engineering, and locally operating feedbacks rather than from sustained external inputs. Examples include the tropical rainforest (Davidson, Detling, and Brown 2012), coral reef (Hughes et al. 2010), and beaver‐engineered wetlands (Hood and Larson 2015). In these systems, ecosystem structure and function persist primarily through endogenous processes, making them strong reference points for high EI.

## 6 Novel Ecosystems

Novel ecosystems arise from historical anthropogenic forcing but are no longer actively managed (Hobbs et al. 2009; Hallett et al. 2013). They self‐organize around altered environmental baselines and operate under feedback that differs fundamentally from historical conditions. Although shaped by legacy effects and altered abiotic or biotic conditions, novel ecosystems retain substantial ecological self‐organization and may converge toward new attractor states under modified baselines. Stability is often transitional or variable during reassembly. Examples include postindustrial forests, abandoned agricultural lands, and urban wildlands. While these systems may retain substantial self‐organizing capacity, their trajectories are constrained by legacy effects and altered abiotic and biotic conditions (Hallett et al. 2013). As a result, some novel ecosystems may retain partial reversibility, whereas others exhibit ecological lock‐in, limiting their capacity to return to historical states.

## 7 Dialectical Ecosystems

Dialectical ecosystems constitute a distinct regime in which ecological self‐organization and human intervention interact dynamically rather than additively (Angeler and Maybee 2025). Their structure, resilience, and trajectories emerge from ongoing tensions between opposing organizational forces, such as productivity versus biodiversity or engineered control versus ecological feedback.

These regimes emerge from dynamic tensions between competing organizational forces such as productivity and biodiversity or engineered control and ecological feedback producing novel attractor configurations. Human organization in such systems is not purely coercive; governance structures, cultural practices, and social learning networks may themselves exhibit adaptive properties. Consequently, ecological and social self‐organization become coupled, generating hybrid regimes with emergent feedback architectures, path dependence, and coevolving social–ecological dynamics.

Thus, dialectical ecosystems may exhibit high but coupled self‐organization structured across interacting domains, often with negotiated or variable stability. Examples include adaptive comanaged landscapes and urban green infrastructures.

## 8 Coerced Regimes

At the opposite end of the spectrum lie coerced regimes, defined as system states actively maintained through sustained external inputs that suppress or override endogenous ecological feedback (Angeler et al. 2020; Sundstorm et al. 2023).

In these systems, ecological feedback is suppressed, overridden, or tightly controlled, creating configurations that are structurally ordered but functionally brittle. Examples include industrial agricultural systems, chemically treated eutrophic lakes, and fire‐suppressed forests (Shimoda and Arhonditsis 2016; Kreider et al. 2024). Apparent stability in coerced regimes is externally enforced rather than internally generated. Adaptive capacity is constrained, and once management pressure is relaxed, rapid reorganization or collapse may occur. Coerced regimes demonstrate that apparent order, productivity, or stability do not necessarily indicate EI, as these properties collapse rapidly once external inputs are removed. As such, coerced regimes should not be classified as ecosystems with moderate or high EI despite their organized appearance.

Accordingly, EI—defined here as the ability of a system to maintain self‐organization (Müller 2005)—is expected to be highest where feedback‐driven organization remains autonomous (wild and many novel systems) and lowest where apparent order depends on continuous coercion and suppression of endogenous feedback (coerced regimes), with dialectical ecosystems reflecting coupled social–ecological organization in which integrity depends on the persistence of adaptive feedback across both domains.

**FIGURE 1:**
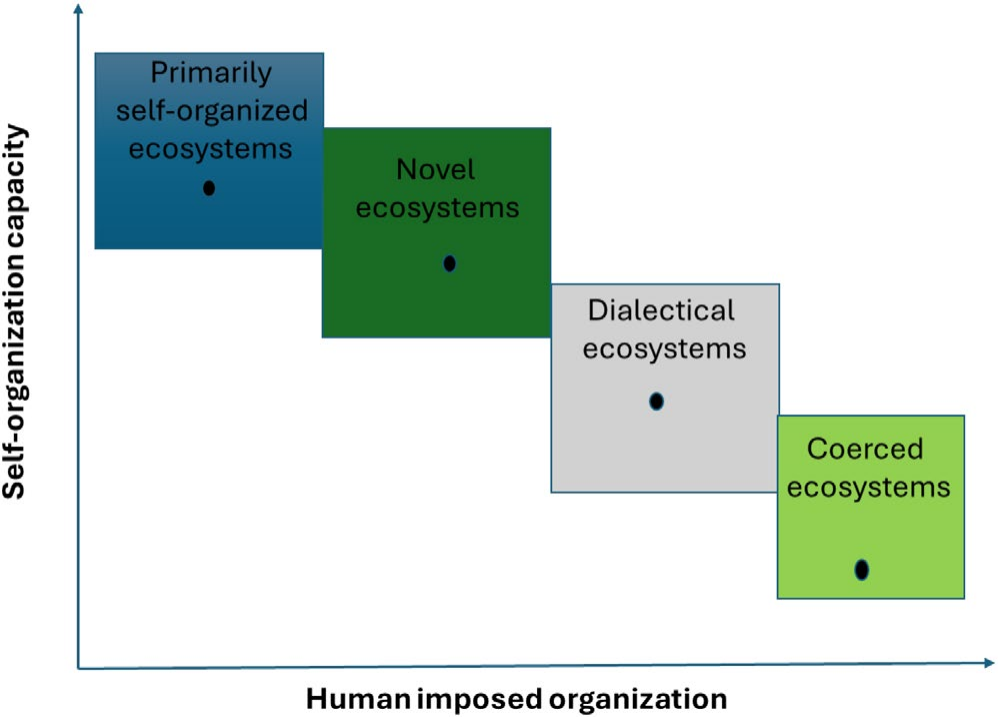
Conceptual placement of ecosystem types along two interacting dimensions: Capacity for self‐organization (vertical axis) and degree of management coercion (horizontal axis). Four zones are depicted: Primarily self‐organized systems, novel ecosystems, dialectical ecosystems, and coerced regimes.

Variety within each of these categories and their trajectories is high. However, one can generalize simply by assessing the self‐organization and stability of characteristics such as primary productivity (Table 1, Figure 1).

Together, this typology provides a framework for evaluating ecosystems not solely by their current configuration, but by the degree to which organization is autonomous rather than externally enforced. Within this perspective, EI is highest where self‐organization is sustained through endogenous ecological feedback, and lowest where order is maintained through continuous coercion. Positioning ecosystems along the self‐organization–coercion axis therefore enables threshold prediction, vulnerability assessment, and adaptive management across increasingly human‐modified landscapes (Table 2).

**TABLE 2:** Relative self‐organization and stability of ecosystem types.

	High self‐organization	Low self‐organization
High stability	Wild systems	Coerced regimes^a^
Variable/transitional stability	Novel ecosystems	Disturbed areas^b^
Coupled/negotiated stability	Dialectical ecosystems	—

^a^ Coerced regimes may exhibit high apparent stability (e.g., productivity), but this stability is externally enforced rather than generated by endogenous ecological feedback.

^b^ Disturbed areas are not examined in this article in the context of hybrid socio‐ecological systems.

## 9 The Role of Self‐Organization in Hybrid Ecosystems' Function, Sustainability, and Integrity in the Anthropocene

In the Anthropocene, hybrid ecosystems, combining elements of self‐organization with human‐imposed structures, are the dominant form of terrestrial ecosystems (Ellis and Ramankutty 2008; Kennedy et al. 2019). Understanding the role of self‐organization in these systems is essential for evaluating their EI, sustainability, and their capacity to maintain critical ecosystem functions under escalating environmental pressures.

Although hybrid ecosystems are impacted by anthropogenic pressures, they continue to rely heavily on self‐organizing dynamics for their resilience and functionality. For example, in croplands, soil microbial cycling and plant‐pollinator networks persist even under intensive agricultural practices, supporting nutrient cycling, pest control, and soil health (Kremen and Miles 2012; Gliessman 2015). In rangelands, spontaneous vegetation pattern formation and grazing feedbacks help maintain forage production and minimize erosion alongside human management (Briske et al. 2023; Sanderson et al. 2020). In village mosaics, agroecological succession and seed exchange networks foster biodiversity, genetic resilience, and ecosystem service provision (Altieri and Toledo 2011; Tittonell 2014).

Self‐organization supports the internal stability of hybrid systems, and also organization increases their capacity to deliver critical ecosystem services, including provisioning services (e.g., food, fiber, and fuel), regulating services (e.g., climate moderation, erosion control), supporting services (e.g., nutrient cycling, habitat provision), and cultural services (e.g., traditional knowledge and identity).

Moreover, self‐organizing processes reduce the dependency of hybrid ecosystems on constant human inputs. Systems that maintain mechanisms such as self‐repair through successional dynamics, feedback stabilization, or trophic redundancy demonstrate greater adaptive capacity and long‐term sustainability (Walker et al. 2004; Chazdon and Guariguata 2016).

However, challenges remain. Hybrid systems are often vulnerable to management practices that suppress self‐organization such as monoculture expansion, urban sprawl, overgrazing, or fire suppression, which can lead to reduced resilience, ecological degradation, and the loss of essential services (Benayas et al. 2009; Kowarik 2011).

Recognizing the intertwined roles of self‐organization and human management offers a practical pathway for adaptive ecosystem management in the Anthropocene. Strategies should not only aim to mitigate human impacts but to enhance and protect self‐organizing processes such as fostering landscape heterogeneity, conserving functional redundancy, and promoting multi‐scale feedbacks (Sayer et al. 2013; Aronson et al. 2017).

In conclusion, self‐organization remains a cornerstone of EI in hybrid ecosystems. These intrinsic processes must be protected and strengthened to maintain ecosystem services, promote resilience, and ensure the sustainability of human‐environment systems in an era of accelerating global change.

## 10 Using Self‐Organization as a Quantitative Measure of Hybrid Ecosystem Integrity

EI is commonly viewed as a measure of ecosystem health (Leopold 1949; Karr 1991). However, its definition, quantification, and application are generally vague. Historically, the concept of EI focused on preserving pristine environments, primarily within protected areas, to maintain what were considered to be untouched, natural conditions (Hermoso and Clavero 2013; Andreasen et al. 2001). Aldo Leopold's seminal work (1949) emphasized the importance of maintaining the integrity of biotic communities to ensure ecosystem stability (Wurzebach and Schultz 2016). His work later became foundational for environmental policies such as the Clean Water Act (Karr 1991; Cafaro and Primack 2014).

As the concept of EI developed, it filled an important gap by describing the capacity of ecosystems to maintain valuable services under both internal and external drivers (Bridgewater et al. 2014). EI has been defined as the ability of ecosystems to support the provision of services in the absence of anthropogenic disturbances (Del Leo and Levin 1997; Müller et al. 2000), or as a “precaution against unspecific ecological risks within the framework of sustainable development” (Karr and Dudley 1981). Scientist later replaced the term “supporting ecosystem services” with EI to describe conditions capable of sustaining ecosystem services (Burkhard et al. 2010; Haines‐Young and Potschin 2010; Kandziora et al. 2013); thus the interpretation of EI has become dependent on societal preferences (Kay and Schneider 1994).

When EI is defined solely as the lack of human interference (Hermoso and Clavero 2013), assessing the actual impact of anthropogenic disturbances on an ecosystem becomes problematic. Linking high EI to pristine conditions creates a circular argument: assessing the impact of anthropogenic activities on an ecosystem using EI is unfeasible when it is measured by the presence or absence of anthropogenic activity. Therefore, a clearer distinction between the presence of anthropogenic activity and its impact is necessary. This is especially relevant when discussing hybrid ecosystems. The processes and structure of a hybrid ecosystem strongly affected by anthropogenic disturbances may still be largely shaped by self‐organization (high EI), or it may be very heavily affected by external forces (low EI).

Thus, we propose an approach that does not focus solely on human interference or needs. Instead, it examines the ecosystem's intrinsic characteristics, particularly its ability to maintain self‐organization. This approach suggests that the integrity of an ecosystem can remain high if it autonomously organizes itself, even when influenced by anthropogenic factors. It follows the definition of EI proposed by Müller (2005), framed as “the ability of a system to maintain self‐organization.”

This definition can be applied to hybrid ecosystems by recognizing that these systems, despite human modification, retain self‐organizing processes that are integral to their functioning. The key advantage of this approach is that it defines EI based on the system's ability to maintain organized, functional processes, irrespective of human impacts. Thus, the anthropogenic impacts are evaluated by the degree to which self‐organizing processes persist, and EI can be graded based on the extent of self‐organization present in a system, even if anthropogenic impacts are evident.

Self‐organization in hybrid ecosystems can be quantified. Using Müller's definition of EI (2005), we can quantify the level of self‐organization in a hybrid system and, by extension, determine its EI. A system that is not pristine but continues to exhibit high levels of self‐organization can still be considered to have high integrity, even with anthropogenic influence. Integrity is determined by the ecosystem's functioning—its ability to maintain core processes such as nutrient cycling, energy flow, and species interactions—rather than by the mere presence of external, human influences.

In conclusion, the definition of EI as “the ability of a system to maintain self‐organization” provides a useful framework for evaluating the integrity of hybrid ecosystems. It shifts the focus from a static, anthropocentric view of EI to one that recognizes the dynamic, self‐organizing nature of ecosystems, even under human influence. This approach allows for more nuanced assessments of ecosystem health, particularly in the hybrid‐ecosystem dominated Anthropocene.

## 11 Framework for Navigating Self‐Organization and Ecological Integrity of Hybrid Ecosystems in the Anthropocene

**FIGURE 2:**
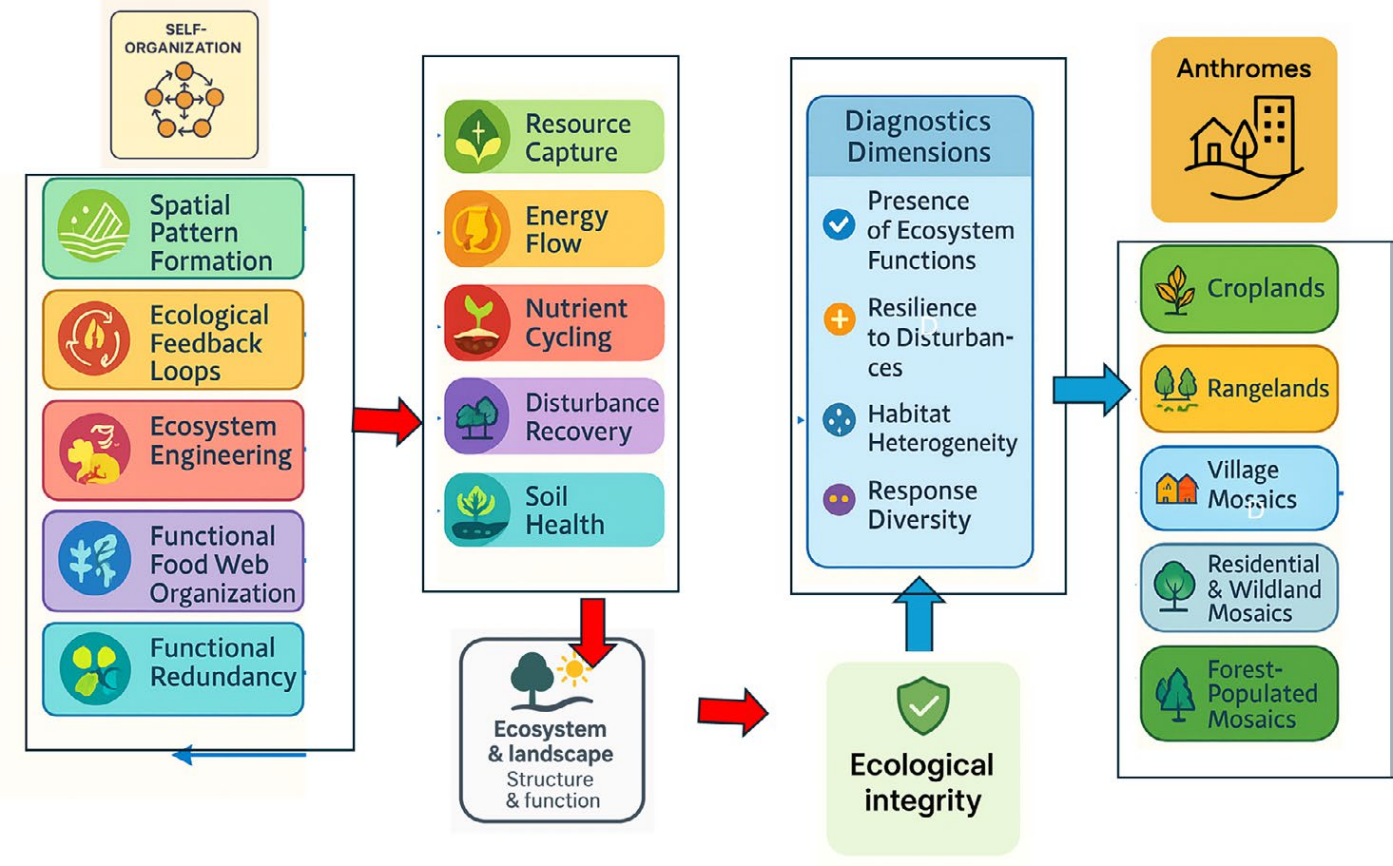
Framework for navigating self‐organization and ecological integrity of hybrid ecosystems in the Anthropocene. This flowchart presents a comprehensive, process‐oriented approach to assessing and managing the EI of hybrid ecosystems. The framework begins by identifying key self‐organizing processes and linking them to core ecosystem functions that regulate ecosystem and landscape structure. The resulting ecosystem properties ultimately determine the ecological integrity of hybrid ecosystems. Key diagnostic dimensions for assessing ecological integrity are also proposed. These navigating processes are mapped to specific anthrome categories, reflecting the diversity of human‐imposed organization within hybrid ecosystems. The framework emphasizes the critical role of self‐organization in maintaining the resilience and functionality of hybrid ecosystems.

Navigating the EI of hybrid ecosystems in the Anthropocene requires a conceptual framework that integrates the role of self‐organization in ecosystem function across different ecosystem types. We propose a roadmap (Figure 2) that identifies key self‐organizing processes, such as spatial pattern formation, population self‐regulation, ecological feedback loops, ecosystem engineering, functional food web organization, and functional redundancy. These self‐organizing processes regulate core ecosystem functions, including resource capture, energy flow, nutrient cycling, disturbance recovery, and soil health. These processes are the foundation of whole ecosystem and landscape properties, which in turn ultimately determine the EI of hybrid ecosystems.

The EI of hybrid ecosystems refers to their ability to maintain essential structure, function, and resilience over time, despite the influences of natural self‐organization and anthropogenic pressures. It reflects the capacity of these systems to sustain ecosystem processes and the services they provide, even under human‐imposed organization. This integrity is determined by the continued presence of self‐organizing processes that interact with and complement human intervention, enabling the system to adapt and persist under changing environmental and socio‐economic conditions.

Building on this roadmap, the framework proposes a targeted evaluation of EI based on four diagnostic dimensions: (1) the presence and redundancy of key ecosystem functions; (2) the system's resilience to disturbances such as drought, fire, or land‐use change; (3) the spatial structure and heterogeneity of vegetation or habitat; and (4) the level of functional and response diversity among biological communities. These criteria offer a multidimensional lens through which the EI of hybrid systems can be assessed and compared.

Importantly, this process‐based approach emphasizes the mechanisms by which self‐organization in hybrid ecosystems maintains their capacity to adapt and persist. This aligns with contemporary paradigms in resilience and sustainability science, which prioritize adaptive capacity, feedback regulation, and functional integrity over time.

By explicitly incorporating self‐organization into assessments of EI, this framework enables managers and policymakers to identify which self‐organization processes and ecosystem functions are most critical to conservation or restoration within specific hybrid ecosystems. In doing so, it promotes strategies that work with, rather than override, the inherent ecological processes of ecosystems. Such strategies are particularly vital in the Anthropocene, when most terrestrial ecosystems are hybrid and face mounting socio‐ecological challenges.

In addition to quantifying self‐organization in an ecosystem, we must quantify the relative impact of each force in an ecosystem, including those driven by self‐organization and those driven by imposed organization. Using the developed quantitative tools, one can assess the integrity of an ecosystem and thus improve management practices. Various conservation tools can be used on hybrid systems to assess their efficiency.

## 12 Assessing Self‐Organization Capacity as a Dimension of Hybrid Ecosystem Integrity

The EI of hybrid ecosystems hinges not only on biodiversity, but also on the systems' internal capacity for self‐organization—the ability to sustain functional complexity through endogenous feedbacks and adaptive responses. In ecosystems shaped by both natural processes and human intervention, this capacity determines whether the characteristics of the ecosystem are maintained autonomously or become dependent on continued external inputs.

To operationalize this concept, we propose a composite framework for estimating self‐organization capacity from measurable ecological indicators spanning structural, functional, and thermodynamic domains. This approach complements existing biodiversity and ecosystem service metrics by capturing latent systemic properties that underpin long‐term resilience and autonomy.

The following section outlines a structured method for inferring self‐organization capacity in hybrid ecosystems, highlighting three foundational indicator domains: (i) network organization; (ii) thermodynamic performance; and (iii) spatial–temporal complexity, alongside integrative variables from the Essential Biodiversity Variables (EBV) framework. Together, these indicators can be combined into a scalable index applicable across biomes, management regimes, and disturbance histories, offering a powerful lens through which to monitor the shifting boundaries of self‐organization and coercion in anthropogenically influenced ecosystems.

## 13 Inferring Ecosystem Self‐Organization Capacity From Ecological Indicators

Ecosystem self‐organization capacity refers to the ability of an ecosystem to autonomously develop, maintain, and adapt its structural and functional complexity through internal feedback and processes, without continuous external inputs (Jørgensen and Müller 2000; Jørgensen et al. 2010). This latent property can be estimated using a composite of measurable indicators across Jørgensen et al. (2010) three domains plus two more domains:

1. Network organization: Metrics such as ascendency, capacity, and overhead derived from ecological network analysis reflect the magnitude and coordination of matter flows within trophic or process networks. Such networks are often indicative of internally organized structures (Ulanowicz 1997; Jørgensen and Müller 2000; Mageau et al. 1995; Patrício et al. 2006; Montoya and Pimm 2006; Bascompte and Jordano 2007).
2. Thermodynamic performance: Indicators such as eco‐exergy, specific exergy, and proxies for entropy production capture how ecosystems use and dissipate energy to build and sustain ordered states and the energy flows (Jørgensen and Nielsen 2007; Holdaway et al. 2010; Maes et al. 2011).
3. Spatial–temporal complexity: Spatial metrics, which focus on the emergence of spatial organization, quantify whether spatial patterns emerge beyond randomness (e.g., autocorrelation and patch structure). Measures of landscape heterogeneity and emergent patterning, including entropy‐based metrics and fractal dimensions from remote sensing time series, reflect the degree of multiscale order (Rietkerk and van de Koppel 2008; Levin 1992; Parrott 2010; O'Neill et al. 1988; Li and Reynolds 1993; Kéfi et al. 2007; Loke and Chisholm 2022).
4. Dynamical indicators of organization and resilience focus on early warning signs and on loss of self‐organization and stability due to perturbation (e.g., recovery rates after disturbance; Sheffer et al. 2009; Dakos et al. 2012).
5. Information‐theoretic measures, which reflect constrained information flow among system components, quantify it using statistical measures to assess nonrandom information flow (Prokopenko et al. 2008; Mayer and Rietkerk 2004).

See examples, description and indicators in Table 3. Self‐organization as a predictor of EI can be validated through experimental frameworks, in which controlled perturbations are applied and ecosystem response is measured. Experiments can be conducted in mesocosms, long‐term ecological monitoring can provide insights, temporal remote‐sensing data can be assessed, and models can provide simulations. Such frameworks can be used to test whether systems exhibiting higher levels of self‐organization better maintain biodiversity, function, or stability under perturbations. Such approaches have already been explored in microbial and plant systems and provide a concrete foundation for validating EI based on ecosystem self‐organization (Drake and Griffen 2010).

**TABLE 3:** Domains and indicators for inferring ecosystem self‐organization capacity.

Domain	Key indicators	Description	Examples in hybrid ecosystems
Network organization	Ascendency, capacity, overhead, cycling index	Reflects matter‐flow coherence and internal feedback structure	Nutrient cycling in agroecological systems, food web stability in reserves
Thermodynamic performance	Eco‐exergy, specific exergy, entropy production proxies (e.g., ET)	Measures system efficiency in energy use and order maintenance	Evapotranspiration efficiency in irrigation mosaics
Spatial–temporal complexity	Entropy metrics, fractal dimension, patch dynamics	Quantifies landscape‐level structural complexity and pattern emergence	Heterogeneous land‐use mosaics, temporal variability in NDVI
Biotic diversity (EBVs)	Flora/fauna diversity, species interactions, phenology	Tracks diversity components supporting redundancy and adaptive capacity	Mixed‐crop landscapes, species turnover in semi‐managed forests
Abiotic heterogeneity	Soil, water and air variables, microclimatic variation	Captures environmental variability crucial for sustaining internal feedbacks	Terraced landscapes, multi‐zone watershed systems
Ecosystem function	Primary/secondary productivity, nutrient retention, disturbance regime	Links functioning to resilience and feedback modulation	Productivity in silvopastoral systems, nutrient retention in riparian zones

*Note:* These domains jointly inform the self‐organization capacity index, offering a comparative basis for assessing resilience, integrity, and tipping potential across hybrid systems globally.

These indicators can be standardized within bioclimatic or anthrome‐specific reference sets using *z*‐scores, percentile ranks, or distance‐from‐reference approaches. When combined into a composite “self‐organization capacity index,” they provide a globally scalable and repeatable measure of EI and autonomy. This index can be used to model hybrid ecosystem states along the coercion–self‐organization spectrum, to identify tipping zones, and to monitor integrity trajectories under changing environmental or management regimes (Angeler et al. 2020).

Additionally, hybrid ecosystems can benefit from the integration of EI indicators such as those shown in the EBV framework (Pereira et al. 2013). Structural components, such as biotic diversity (flora, fauna, and habitat), abiotic heterogeneity (soil, water, and air), and process‐level indicators (energy, matter, and water budgets), directly relate to ecosystem self‐organization. EBVs such as species abundance, species interactions, primary productivity, and nutrient retention not only reflect biodiversity patterns but also capture emergent feedback essential for hybrid system functioning. Applying these indicators within hybrid systems can illuminate the codependence of ecological structure and management inputs, helping to detect shifts in self‐organization capacity and to inform adaptive management strategies.

## 14 Conclusions

The profound human‐driven transformations characteristics of the Anthropocene have led to the world‐wide dominance of hybrid ecosystems, in which natural self‐organization processes interact with human‐imposed systems. These hybrid ecosystems play a critical role in sustaining ecosystem functions and human well‐being. This paper demonstrates that self‐organization is a fundamental driver of EI in hybrid ecosystems. It identifies and analyses core self‐organizing processes—specifically, spatial pattern formation, population self‐regulation, ecological feedback loops, ecosystem engineering, functional food web organization, and functional redundancy. We show how these processes underpin essential ecosystem functions across various hybrid ecosystem (anthrome) categories.

We conclude that understanding the degree and nature of self‐organization within hybrid ecosystems is essential for assessing their EI and sustainability. The proposed conceptual framework for evaluating EI integrates these self‐organizing processes, mapping their contributions to hybrid ecosystem structure and function. We argue that EI in these ecosystems should not be defined solely by the extent of human impact, but by the continued presence and interaction of self‐organizing processes with human interventions (Figure 2).

Moreover, the framework provides a multidimensional approach to assessing EI, emphasizing diagnostic criteria such as the redundancy of key ecosystem functions, resilience to disturbances, spatial structure, and functional and response diversity among biological communities. These dimensions allow for context‐sensitive assessments of hybrid ecosystems and offer a dynamic, process‐based approach for monitoring their integrity. Self‐organization can be useful in assessing EI in all hybrid ecosystems, especially when the self‐organization processes are quantified in hybrid ecosystems.

The importance of this framework lies in its ability to guide adaptive management strategies that enhance the inherent ecological processes of hybrid ecosystems. In an era of rapid environmental change, fostering resilience through self‐organization is essential for ensuring the long‐term sustainability of ecosystems and the continued provision of ecosystem services. In conclusion, the role of self‐organization in maintaining EI of hybrid ecosystems is crucial, and acknowledging and reinforcing these processes is vital for promoting sustainability in the Anthropocene.

## Author Contributions

**Elli Groner:** conceptualization (equal), funding acquisition (lead), project administration (lead). **Aviva Peeters:** formal analysis (lead), funding acquisition (supporting), methodology (lead). **Moshe Shachak:** conceptualization (equal), funding acquisition (supporting).

## Funding

This work was supported by Israel Academy of Sciences and Humanities, 964/14; Israel Ministry of Innovation, Science and Technology.

## Conflicts of Interest

The authors declare no conflicts of interest.

## Data Availability

The authors have nothing to report.
